# Two Chinese patients of sporadic Creutzfeldt–Jacob disease with a S97N mutation in *PRNP* gene

**DOI:** 10.1080/19336896.2023.2276921

**Published:** 2023-11-14

**Authors:** Dong-Lin Liang, Qi Shi, Kang Xiao, Ruhan A, Wei Zhou, Xiao-Ping Dong

**Affiliations:** a National Key-Laboratory of Intelligent Tracking and Forecasting for Infectious Disease, NHC Key Laboratory of Medical Virology and Viral Diseases, National Institute for Viral Disease Control and Prevention, Chinese Center for Disease Control and Prevention, Beijing, China; b China Academy of Chinese Medical Sciences, Beijing, China; c Center for Biosafety Mega-Science, Chinese Academy of Sciences, Wuhan, China; d Shanghai Institute of Infectious Disease and Biosafety, Shanghai, China

**Keywords:** CJD, prion, *PRNP*, S97N mutation

## Abstract

Worldwide, 10–15% human prion disease are genetic and inherited, due to the special mutations or insertions in *PRNP* gene. Herein, we reported two Chinese patients with rapidly progressive dementia who were referred to the national Creutzfeldt–Jacob disease (CJD) surveillance as suspected CJD. Those two patients displayed sporadic CJD (sCJD)-like clinical phenotype, e.g. rapidly progressive dementia, visional and mental problems, sCJD-associated abnormalities in MRI. A missense mutation was identified in one *PRNP* allele of these two patients, resulting in a change from serine to asparagine at codon 97 (S97N). RT-QuIC of the cerebrospinal fluid samples from those two cases were positive. It indicates that they are very likely to be prion disease.

## Introduction

Human prion diseases include Creutzfeldt–Jakob disease (CJD), Gerstmann–Straussler–Scheinker syndrome, fatal familial insomnia (FFI) and Kuru, which can be classified as sporadic, inherited and acquired. Approximately, 10–15% of human prion diseases are inherited closely related with dozens of the point mutations or the insertions in the octarepeat region within *PRNP* gene encoding for PrP protein [1–3]. About 20 different types of *PRNP* mutations have been reported in Chinese patients with prion diseases, among them T188K genetic CJD (gCJD), D178N FFI and E200K gCJD are the most frequent [4]. In this report, we described two unrelated Chinese patients with progressive dementia, who displayed sporadic CJD (sCJD)-like clinical progressions. A point mutation was identified in one allele of *PRNP* gene of those two cases, leading to an exchange from serine (S) to asparagine (N) at codon 97 (S97N).

### Case presentation

Patient A, 62-year-old male driver, was admitted to the hospital due to acute memory loss and slurred speech for half month. The patient began to experience memory loss half month ago without obvious incentives, manifested as recent memory loss, unresponsiveness, forgetting things, accompanied by slurred language, difficulty finding words, unsteady walking, and incoordination of limbs. These symptoms aggravated and affected his daily life gradually. Occasionally, he displayed choking when drinking. Brain MRI and CT scanning in the local hospital ruled out acute cerebrovascular disease. With the diagnosis of dementia, he was treated with neutrophic nerve growth factor; however, the symptoms were still aggravating. He was further transferred to Nanjing Brain Hospital and considered as cognitive impairment. Other symptoms appeared gradually, such as involuntary movements of limbs during sleep, unconsciousness disturbance, dizziness, numbness of limbs, dysphagia, less food intake, vomiting yellow liquid when getting up. Head MRI showed high signal in caudate nucleus and putamen and asymmetrical cortical ribbon in DWI. Lumbar puncture cerebrospinal fluid (CSF) biochemistry was in the normal ranges, with the pressure of 70 mmH_2_O and protein of 0.33 g/L (reference range 0.15–0.45 g/L). He received three doses of COVID-19 inactivated vaccine without adverse reaction, and he was free of COVID-19.

Review of the past medical history did not find special significance. He had benign prostatic hyperplasia. Several years ago, he had cholecystectomy because of gallbladder stones and had a suturing surgery because of right ear trauma. He denied blood transfusion and food or drug allergy history. There was no similar neurological disease or other hereditary diseases in three generations of two lines of his family. He died approximately 6 months after onset (from March to September 2022). No brain post-mortem was performed.

Patient B, an 82-year-old female, was admitted to the hospital because of sudden dizziness and unsteady walking for 2 months. The patient complained sudden dizziness without obvious incentives that aggravated when the body position changed. Subsequently, unsteady walking and auditory hallucinations were noticed. She went to the General Hospital of the Eastern Theater Command with the suspect of cerebral infarction. Physical examination identified staggering gait and uncooperative in physical examination, with blood pressure of 152/74 mmHg. Neurological examination revealed clear mind but unresponsive, poorly fluent speech, decreased cognitive functions such as comprehension and memory. Horizontal nystagmus was recorded in both eyes. Bilateral finger-to-nose and heel-knee-shin tests were uncompleted. Walking in a straight line was difficult. Brain MRI showed subacute infarction in the right temporal lobe and multiple lacunar infarctions in the white matter of bilateral lateral ventricles and frontal and parietal lobes. High signal in caudate nucleus and putamen and asymmetrical cortical ribbon syndrome in DWI were recorded. CSF biochemistry was in the normal ranges, with the pressure of 120 mmH_2_O and the protein content of 0.41 g/L. She did not receive COVID-19 vaccine and was free of COVID-19.

She had diabetes for many years. There was no similar disease or other hereditary diseases in three generations two lines of the family. She died about 70 days after the onset of symptoms (from January to March 2022). No brain post-mortem was performed.

### Laboratory assays

**Figure 1.**
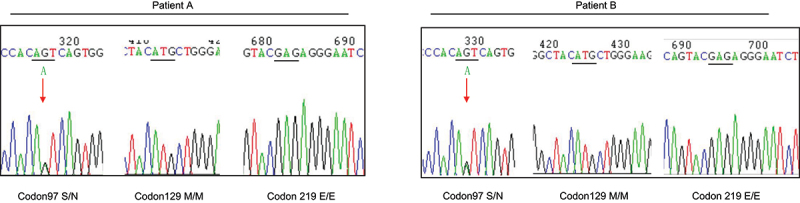
Graphic presentation of the sequencing analysis of *PRNP*. DNA sequences from the two patients at codon 97, codon 129 and codon 219. Heterozygous transition at codon 97 from ser (S) to asp (N) in one *PRNP* allele (left graph), homozygous methionine (M) at codon 129 (middle graph) and homozygous glutamic acid (E) at codon 219 (right graph). Arrow indicates the heterozygote of S97N.

Western blot for CSF 14-3-3 protein was performed. Specific bands mobilizing roughly at 28 kDa were detected in the CSF sample of patient B but not in CSF of patient A. The genomic DNAs were extracted from the peripheral blood leukocytes utilizing Qiagen’s DNA purification kit. After *PRNP* specific PCR amplification, the products were employed direct sequencing. A missense mutation was identified in one *PRNP* allele of these two patients, resulting in a change from serine to asparagine at codon 97 (S97N, Figure 1). The polymorphisms of codon 129 and codon 219 of those two patients were methionine homozygote (M129M) and glutamic acid homozygote (E219E). There was no other mutation in the rest of *PRNP* sequences. The family members and other relatives were reluctant to donate blood samples for *PRNP* sequencing.

**Figure 2.**
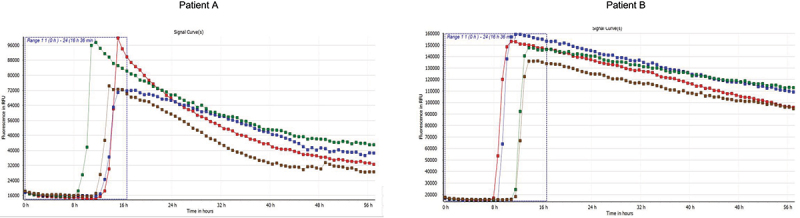
The spectra of CSF RT-QuIC assays. A 15 µl CSF sample was added to 85 µl of reaction mixture containing 1 mg/mL rHarPrP 90–231 into the each well of a 96 well plate. The assays were conducted in FLUOstar OMEGA plate reader (BMG Labtech, Germany). Each sample consists of four replicates (wells). Y-axis represents relative fluorescent unit (rfu, ThT value). X-axis represents the time (h) post-reaction.

CSF RT-QuIC assays were conducted according to the protocol described previously [5], using recombinant truncated hamster PrP (HaPrP90–231) as the substrates. Positive reactions in CSF RT-QuIC were detected in those two patients. The lag times of those two cases were comparable within 16 h post-reaction and the values of relative fluorescent unit (rfu) of patient B were higher than that of patient A (Figure 2).

## Discussion

Here, we have reported two Chinese patients with progressive dementia and sCJD-like clinical manifestations who contain an S97N mutation in their *PRNP* genes. Those two cases were suspected as sCJD before *PRNP* sequencing. Reviewing the literatures indicates that the two cases in this report are the 2^nd^ and 3^rd^ dementia patients with S97N substitution in the *PRNP* gene. The first dementia case with S97N substitution was also a Chinese female, who displayed progressive dementia, mental problems and vision disturbance at the age of 74 [6]. Her clinical diagnosis was probable Alzheimer disease with the duration of more than 3 years. Contrary to the 1^st^ S97N case, two cases here showed sCJD-like clinical phenotype, sCJD-associated abnormalities in MRI, and more importantly, positive in CSF RT-QuIC. The clinical durations of those two patients were much shorter than the 1^st^ case, being 6 and 3 months after onset, respectively. Despite lacking neuropathological evidence, the diagnoses of probable sCJD with S97N substitution for these two cases are largely accurate.

Using some available databases with Asian population as the cohort, we have evaluated the frequency of S97N mutation in general population. Searching in the database of ChinaMap (http://www.mbiobank.com/) with Chinese population as the cohort identified that the frequency of S97N (rs56362942, chr20:4699510 (GRCh38.p14)) is 0.00122781 (26/21176), whilst S97N frequency is 0.0003 (1/2922) in the database of KRGBD (https://gnomad.broadinstitute.org/) with Korean population as the cohort and 0.0001387 (2/14420) in the database of gnomAD (https://gnomad.broadinstitute.org/) with the other Asian population as the cohort. Apparently, the frequency of S97N in Chinese is higher than the other Asian races. Calculation of the ratio of S97N cases out of 2143 diagnosed PrD cases (including sCJD and gPrD) from 2006 to 2021 [7] and 214 PrD cases in 2022 (unpublished data) in our surveillance system shows quite comparable frequency (0.0012728, 3/2357) as general Chinese population. Those data may indicate that S97N substitution in *PRNP* is likely a rare polymorphism.

Because of lacking brain post-mortem of these two CJD cases, whether S97N substitution has the potential to influence the neuropathology and PrP^Sc^ biochemistry remains unknown. Meanwhile, whether S97N substitution affects the susceptibility, clinical features and progression of CJD is hard to address right now, simply due to the limited case number. Although the S97N mutation is more like as a rare polymorphism, the exact significance in the pathogenicity of prion disease and other neurodegenerative diseases still needs further study.
